# Facial emotion recognition in agenesis of the corpus callosum

**DOI:** 10.1186/1866-1955-6-32

**Published:** 2014-08-14

**Authors:** Matthew W Bridgman, Warren S Brown, Michael L Spezio, Matthew K Leonard, Ralph Adolphs, Lynn K Paul

**Affiliations:** 1 DuBois Regional Medical Center, 15801 DuBois, PA, USA; 2 Travis Research Institute, Fuller Theological Seminary, 91101 Pasadena, CA, USA; 3 Division of Humanities and Social Sciences, Caltech, 91125 Pasadena, CA, USA; 4 Scripps College, 91711 Pomona, CA, USA; 5 Neurological Surgery, University of California, 94117-1080 San Francisco, CA, USA; 6 Division of Biology, Caltech, 91125 Pasadena, CA, USA

**Keywords:** Corpus callosum agenesis, Corpus callosum, Facial emotion

## Abstract

**Background:**

Impaired social functioning is a common symptom of individuals with developmental disruptions in callosal connectivity. Among these developmental conditions, agenesis of the corpus callosum provides the most extreme and clearly identifiable example of callosal disconnection. To date, deficits in nonliteral language comprehension, humor, theory of mind, and social reasoning have been documented in agenesis of the corpus callosum. Here, we examined a basic social ability as yet not investigated in this population: recognition of facial emotion and its association with social gaze.

**Methods:**

Nine individuals with callosal agenesis and nine matched controls completed four tasks involving emotional faces: emotion recognition from upright and inverted faces, gender recognition, and passive viewing. Eye-tracking data were collected concurrently on all four tasks and analyzed according to designated facial regions of interest.

**Results:**

Individuals with callosal agenesis exhibited impairments in recognizing emotions from upright faces, in particular lower accuracy for fear and anger, and these impairments were directly associated with diminished attention to the eye region. The callosal agenesis group exhibited greater consistency in emotion recognition across conditions (upright vs. inverted), with poorest performance for fear identification in both conditions. The callosal agenesis group also had atypical facial scanning (lower fractional dwell time in the eye region) during gender naming and passive viewing of faces, but they did not differ from controls on gender naming performance. The pattern of results did not differ when taking into account full-scale intelligence quotient or presence of autism spectrum symptoms.

**Conclusions:**

Agenesis of the corpus callosum results in a pattern of atypical facial scanning characterized by diminished attention to the eyes. This pattern suggests that reduced callosal connectivity may contribute to the development and maintenance of emotion processing deficits involving reduced attention to others' eyes.

## Background

Individuals with agenesis of the corpus callosum (AgCC) offer unique insights regarding the cognitive skills that depend specifically upon callosal connectivity. The corpus callosum (CC), the bundle of approximately 200 million axons [1] that interconnect the two cerebral hemispheres is the human brain's largest white matter tract. Individuals with AgCC and normal intellectual function have a higher than normal likelihood of displaying social and communication deficits consistent with an autism diagnosis [2-6]. Likewise, reductions in structural [7-10] and functional [11] interhemispheric connectivity via the corpus callosum have been reported in individuals with autism spectrum disorders. As a further window into social cognition in AgCC and also to enable direct comparisons with autism, we studied emotion recognition and eye fixations from facial expressions, processes known to be impaired in autism [12,13].

Undoubtedly, social interactions pose some of the most complex cognitive challenges in adult life and are a significant area of concern for individuals with AgCC. Neuropsychological studies of AgCC highlight a pattern of deficits in problem solving, processing speed, and the social pragmatics of language and communication—all of which may contribute to impairments in daily social interactions. Deficits are evident in the comprehension of linguistic pragmatics (including idioms, proverbs, and vocal prosody) [14-17] and in phonological processing and rhyming [14,15,18,19]. There is also evidence of poor comprehension of humor and nonliteral language forms due to a bias toward literal interpretation and difficulty using context to infer meaning [16,20,21]. This impairment in making accurate second-order interpretations of linguistic information is a significant factor in the social profile of AgCC which includes poor conversation skills and restricted verbal expression of emotional experience (similar to alexithymia) [22,23], emotionally and logically impoverished verbal interpretation of social scenes [24,25], and impaired theory of mind in interpreting complex social interactions [26].

The specific neural mechanisms behind the social impairments in AgCC are unclear. It is possible that the social language deficits are a direct result of impaired coordination of the dual language pathways. According to the dynamic dual pathway model of language, the processing of syntax and narrowly construed semantics is lateralized to the left hemisphere, and processing of emotional tone, prosody, and wider semantics associations to the right hemisphere [27-31]. In this model, the corpus callosum is critical for the coordination of this lateralized information and callosal absence would result in particular deficits in processing syntactic and prosodic information [28-30], the very areas of linguistic weakness evident in AgCC. Applied more broadly, this model fits with the right hemisphere model of emotion processing and may account for other aspects of social impairment as well. For example, since the right hemisphere is dominant for emotion perception in both facial and lexical channels [32,33], the dual-pathway model would predict impaired emotion recognition in faces with intact lexical labeling (e.g., gender recognition). There are, of course, individual differences in degree of lateralization of both language and emotion functions [34-36], but the above scheme would apply in general terms across a sample of individuals.

As a consequence of fundamental deficits in information processing and integration, individuals with AgCC may not perceive and comprehend important second-order aspects of social situations, adopting a piecemeal strategy in processing this form of complex information. An emphasis on detail over whole is a cognitive style also seen in autism [6,37,38]. The actual frequency of psychiatric diagnoses in AgCC is unknown; however, surveys completed by caregivers of 720 children and adults with AgCC indicated that approximately 10% of the individuals with AgCC had an autism spectrum diagnosis [39]. In a more recent study, Autism Quotient questionnaires completed by parents of 106 individuals with AgCC indicated that 45% of children, 35% of adolescents, and 18% of adults met the screening criteria for autism [2]. Similarly, in another sample of children with AgCC aged 6 to 11, parent reports indicated significant symptomatic overlap with the autism spectrum [3]. Finally, in the most rigorous diagnostic study to date, the Autism Diagnostic Observation Scales and clinical interviews were conducted in a sample of 26 adults with AgCC; we have found that 30% of individuals met the criteria for an autism spectrum diagnosis based on current symptoms [6].

The present study examines the impact of AgCC on cognitive processing of static faces. Several studies have found reliable, but weak, deficits of individuals with autism spectrum disorder (ASD) in the ability to recognize emotions from facial expressions [12,40-43] (for review, see [44]). The recognition of more complex mental states from faces may show a more reliable impairment in ASD, particularly if only the eye region of faces is shown [45]. There is also evidence that they have an atypical approach to visual scanning of faces which involves allocating less time to looking at eyes and a greater proportion of time to looking at others' mouths [12,46-48]. In light of previous studies of AgCC examining vocal prosody, nonliteral language, and humor as well as the apparent overlap with autism spectrum symptomology, we predicted that participants with AgCC would perform poorly in identifying emotions from faces but perform normally in identifying gender. Additionally, given the phenotypic similarities between AgCC and autism, we expected to see similar patterns of atypical gaze to the face, including reduced gaze to the eyes and increased focus on the mouth.

## Methods

### Participants

Participants included 9 adults with AgCC (7 males, mean age = 28.22 ± 7.34) and 9 adult controls (all male, mean age = 34.33 ± 7.75). Groups were matched with respect to age *t*(15.88) = -1.49, *p* = 0.16, and each group included 2 left-handed individuals (as measured by the short Edinburgh Handedness Questionnaires [49]). Full-scale intelligence quotient (FSIQ) was higher in the control group (112.22 ± 8.21) than the AgCC group (98.22 ± 11.55; *t*(13) = 2.96, *p* < 0.01); however, the groups did not differ on education level (Fisher’s exact *p* = 0.091). Adult participants read and signed an informed consent form before testing in accordance with a protocol approved by the IRB of the California Institute of Technology.

Participants with AgCC were recruited through the National Organization for Disorders of the Corpus Callosum, and control participants were recruited through the use of a classified advertisement posted online. Exclusionary criteria for both groups were as follows: (a) English as a second language; (b) FSIQ of less than 75; (c) history of major head trauma, neurosurgery, or major CNS disorder not associated with AgCC; (d) comorbidity with a persistent seizure disorder; (e) moderate to severe psychopathology; and (f) currently taking psychotropic medications that might have significantly altered test performance. Participants with AgCC were included if they had structural findings that commonly co-occur with AgCC: colpocephaly, Probst bundles, interhemispheric cysts, and occasional small heterotopias. Potential participants with other structural brain abnormalities were not included. The presence of anterior commissure was confirmed in all participants with AgCC. We reviewed the MRI data from all participants with AgCC to confirm that they met these criteria. One of the participants with AgCC had a history of seizure disorder, which was well controlled using a standard therapeutic dose of Depakote. Clinical history suggested the possibility of anxiety and depression in one participant with AgCC and reading disability in two other participants with AgCC. Due to a mechanical error, we had to exclude the eye movement data recorded during emotion recognition with inverted faces for one participant with AgCC.

### Measures

The EyeLink II head-mounted eye-tracking system (SR Research, Hamilton, Ontario) was used to track the eye movements and fixations of participants. This system monitors corneal reflection to map the location, duration, and chronological order of fixations for each stimulus presentation, recording at 250 Hz. Tasks were run on Microsoft Windows XP in Matlab (Mathworks, Inc., Natick, MA, USA) using the Psychophysics Toolbox [50,51] and the EyeLink Toolbox [52].

Task stimuli were taken from the *Pictures of Facial Affect*[53]. The stimulus set includes 46 pictures: 6 pictures of each of 6 emotions (happy, sad, afraid, surprised, angry, disgusted), plus 10 pictures of neutral expressions. Stimuli were balanced for gender. General intelligence was measured in the AgCC group using the Wechsler Adult Intelligence Scale III. Due to time constraints in testing, control participants were given the Wechsler Abbreviated Scale of Intelligence [54].

### Procedures

Participants sat 31 in. in front of a computer screen, wearing the head-mounted eye-tracking system, and were asked to observe pictures of faces presented one at a time on the computer screen. Before the task, the subject's gaze was calibrated and validated (with accuracy better than 0.5° of visual angle for most participants), and before each trial, measurement was corrected for drift due to subtle shifts in head movement.

Four separate tasks were administered in the following order: emotion recognition of upright faces, emotion recognition of inverted faces, gender recognition, and passive viewing. This order reduced the possibility that our primary measure of interest, emotion recognition of upright faces, would be contaminated by previous exposure or familiarity effects. For all tasks, participants were instructed to look at the image normally and then give ratings as described below. For the two emotion recognition tasks (upright and inverted), each run consisted of 46 pictures presented for 1 s each in randomized order, and each picture was followed by a list of 6 emotion words (angry, disgusted, fearful, happy, sad, and surprised) and ‘neutral.’ While looking at the word list, subjects verbally labeled the emotion of the face and the examiner recorded the verbal response. Two runs were administered sequentially, yielding a total of 92 trials for each task. Images (512 w × 768 h) were normalized for overall intensity and centrally displayed using a monitor resolution of 1,280 w × 1,024 h (pixel units) on a 15.9 in. w × 11.9 in. h monitor, at an eye-to-screen distance of approximately 31 in., thus subtending approximately 11° of horizontal visual angle.

For the gender recognition task, the same 46 pictures were presented in randomized order. Participants indicated gender judgment with a key press, which triggered removal of the image. If the participant did not respond by 1 s, the image was removed from the screen and the next was not presented until a response was given.

Finally, for the passive viewing task, participants were instructed simply to look at each of the pictures. Only 1 block of 46 pictures was administered. Because it is uncommon in daily life to see faces for only 1 s at a time, for the passive viewing task, image presentation was extended to 4 s to reduce potential impact of time pressure and encourage a more natural gaze pattern. However, analyses included gaze data from the first second only for comparison with the other tasks.

During each of the four tasks, the eye-tracking system recorded the location and duration of the participant’s fixations, chronological order of fixations, and total number of fixations per region of interest (ROI) in the face. Participants completed the tasks in a small office with no windows and dim lighting for better visibility of the computer screen. Along with the participant, a research assistant was always present in the room.

### Data analyses

**Figure 1:**
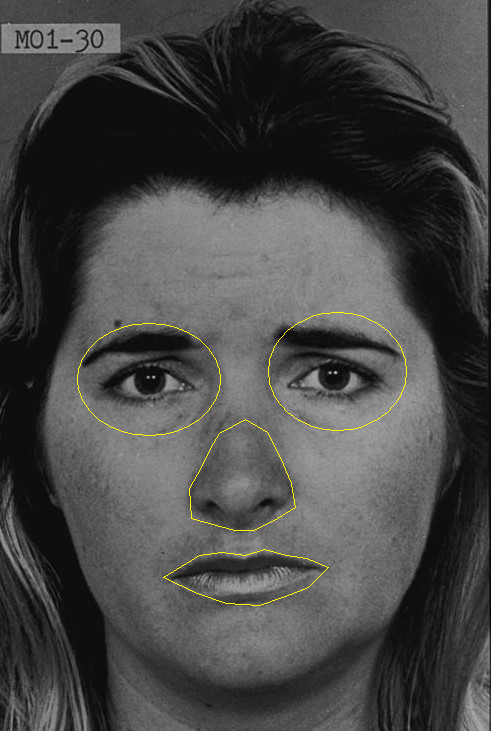
**Sample Ekman image overlaid with region of interest map used in eye-tracking analyses*.*** Modified from *Pictures of Facial Affect*[53] with permission from Paul Ekman Group.

Eye-tracking data were analyzed for fixations using the EyeLink Data Viewer (SR Research, Hamilton, Ontario, Canada). In discriminating fixations, we set saccade velocity, acceleration, and motion thresholds to 30°/s, 9,500°/s^2^, and 0.15°, respectively. Measures of face gaze included fixation number (i.e., the total number of fixations within an area, independent of previous fixation area) and fractional dwell time (i.e., the time during a given trial spent fixating a given area divided by the total time between image onset and response). For the passive viewing condition, analyses were conducted with data from the first second of stimulus presentation only. All analyses included only fixations beginning 50 ms or more after an image appeared and ending at image offset.Regions of interest were drawn for each facial image based on the examiners’ judgment (LKP and MWB), using the drawing functions within EyeLink Data Viewer. We used four ROIs in all: eye region (including both the left and right eyes and the eye sockets around them), nose, mouth, and face. See Figure 1 for a visual depiction of regions.

Fixations falling within the ROIs, but not on ROI edges, were included for the ROI-specific analyses. The proportion of time spent fixating a given ROI, fractional dwell time, was calculated by summing the fixation durations within that ROI and dividing by the total trial time. Fractional dwell times within the eye and mouth regions were further normalized by the face-specific fractional dwell time.

Repeated-measures ANOVA was used to analyze the accuracy data for both emotion recognition tasks (2 groups by 6 emotions, with and without inclusion of neutral trials) as well as eye-tracking data—number of fixations and fractional dwell time (2 groups by 3 ROIs: eye region, nose, mouth). *T* tests were used for post hoc analyses.

Three additional procedures were implemented to address group differences in FSIQ. First, we report the effect of FSIQ on all measures for each group. Second, all analyses (accuracy and eye-tracking) were repeated with introduction of FSIQ as a covariate. Mauchly’s test of sphericity was conducted for each ANCOVA, and in cases where sphericity was violated at *p* < 0.05, the Huynh-Feldt correction was used if epsilon was greater than or equal to 0.75 and Greenhouse-Geisser was used if epsilon was less than 0.75. Significant findings in ANCOVA were followed by post hoc comparisons using univariate ANCOVA with significance set at *α* = 0.05. For the upright-face emotion recognition task, we compared groups on latency to first fixation within the eye region and on percentage of trials in which first fixation fell within eye region. Finally, for each group, we conducted point-biserial correlations between accuracy on upright emotion-recognition task and two eye-tracking variables: latency to fixate within eye region accuracy and dwell time within eye regions. The third additional procedure involved repeating all analyses using IQ-matched groups comprised of 7 individuals from each group (FSIQ: AgCC 102.71 ± 8.44; control 110.14 ± 8.15), who were also matched on age (AgCC 26.71 ± 5.47; control 32.00 ± 7.02) and education (Fisher’s exact *p* = 0.19).

## Results

### Emotion recognition: upright faces

**Figure 2:**
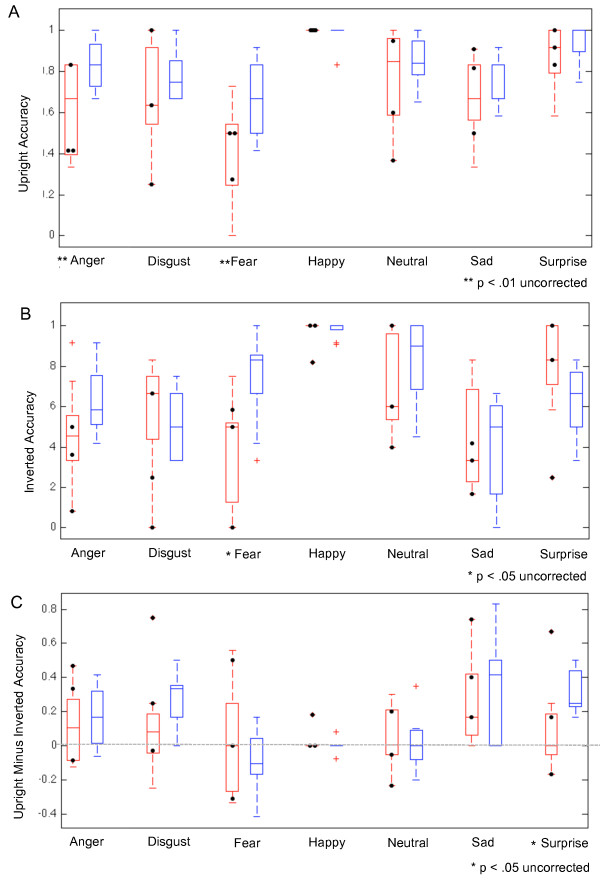
**Emotion recognition accuracy by group and stimulus emotion.** Accuracy of emotion recognition for upright faces **(A)**, inverted faces **(B)**, and the difference between correct recognitions for upright minus inverted faces **(C)**. AgCC group in *red* and control group in *blue. Black circles* mark the accuracy of individuals with AgCC who met current criteria for autism spectrum.

The AgCC group was less accurate than control group in recognizing all 6 emotions (AgCC = 70.65% ± 10.10; control = 81.89% ± 2.77; *F*(1,16) = 10.37, *p* = 0.005, *η*^2^_*p*_ = 0.39; Table 1, Figure 2a), but there was not an interaction of group-by-emotion. Results remained the same when neutral faces were included in the analyses with the 6 emotions (7-emotion-by-2-group ANOVA; Table 1, Figure 2).

**Table 1:** Repeated measures ANOVAs for accuracy of emotion naming

	**ANOVA**	**ANCOVA**	**FSIQ-matched**
	**( *df *) *F*: *p*, *η***^**2**^_***p***_	**( *df *) *F*: *p*, *η***^**2**^_***p***_	**( *df *) *F*: *p*, *η***^**2**^_***p***_
Upright faces: accuracy for 6 emotions			
Group	(1,16) 10.37: 0.005, 0.39	(1,15) 4.57: 0.049, 0.23	(1,72) 8.02: 0.006, 0.10
Emotion	(5,80) 19.39: 0.000, 0.55	(5,75) 1.77: 0.13, 0.12	(5,72) 13.43: < 0.001, 0.48
Interaction	(5,80) 2.07: 0.078, 0.11	(5,75) 1.40: 0.23, 0.09	(5,72) 1.80: 0.13, 0.11
Upright faces: accuracy for 7 emotions			
Group	(1,16) 8.76, 0.009, 0.35	(1,15) 2.76: 0.12, 0.16	(1,84) 7.24: 0.009, 0.079
Emotion	(6,96) 16.59, 0.000, 0.51	(6,90) 2.06: 0.07, 0.12	(6,84) 11.64: *p* < 0.001, 0.45
Interaction	(6,96) 1.78, 0.11, 0.10	(6,90) 1.60: 0.15, 0.098	(6,84) 1.68: 0.14, 0.11
Inverted faces: accuracy for 6 emotions			
Group	(1,16) 0.94: 35, 0.055	(1,15) 0.378: 0.55, 0.025	(1,72) 0.39: 0.53, 0.005
Emotion	(3.3,53.1) 18.07: 0.000, 0.53	(5,75) 1.28: 0.28, 0.078	(5,72) 13.22: < 0.001, 0.49
Interaction	(3.3,53.1) 4.11: 0.009, 0.20	(5,75) 4.97: 0.001, 0.25	(5,72) 5.38: < 0.001, 0.27
Inverted faces: accuracy for 7 emotions			
Group	(1,16) 1.44: 0.25, 0.08	(1,15) 0.55: 0.47, 0.035	(6,84) 0.93: 0.34, 0.011
Emotion	(6,96) 16.63: 0.000, 0.51	(6,90) 1.05: 0.40, 0.07	(6,84) 11.10: < 0.001, 0.25
Interaction	(6,96) 3.53: 0.003, 0.18	(6,90) 4.12: 0.001, 0.22	(6,84) 4.40: < 0.001, 0.24
Upright accuracy minus inverted accuracy for 6 emotions			
Group	(1,16) 2.07: 0.17, 0.12	(1,15) 0.42: 0.53, 0.027	(1,72) 2.52: 0.12, 0.034
Emotion	(3.6,57.3) 5.63: 0.001, 0.26	(5,75) 2.64: 0.03, 0.15	(5,72) 3.49: 0.007, 0.19
Interaction	(3.6,57.3) 1.47: 0.23, 0.08	(5,75) 2.73: 0.03, 0.15	(5,72) 2.38: 0.047, 0.14
Upright accuracy minus inverted accuracy for 7 emotions			
Group	(1,16) 1.73: 0.21, 0.10	(1,15) 0.07: 0.80, 0.005	(1,84) 1.36: 0.25, 0.016
Emotion	(4.3,69.2) 5.33: 0.001, 0.25	(4.4,65.96) 2.35: 0.06, 0.14	(6,84) 3.20: 0.007, 0.19
Interaction	(4.3,69.2) 1.44: 0.23, 0.08	(4.4,65.96) 2.58: 0.04, 0.15	(6,84) 2.48: 0.03, 0.15

FSIQ was correlated with accuracy of emotion recognition in the control group (7 emotions *r* = 0.76, *p* = 0.02, 95% confidence interval (CI) 0.20 to 0.95; 6 emotions *f* = 0.71, *p* = 0.03, 95% CI 0.08 to 0.93; Additional file 1: Table S1). However, FSIQ did not predict emotion recognition in the AgCC group.

In the 6-emotion-by-2-group ANCOVA (Table 1), the AgCC group remained less accurate than the control group (*F*(1,15) 4.57, *p* = 0.049, *η*^2^_*p*_ = 0.23), and there was not an interaction of group-by-emotion. Including neutral in the ANCOVA analyses reduced the effect size of difference between groups, but the pattern remained consistent (AgCC = 71.70% ± 10.11; control = 82.37% ± 3.83). It must be noted that this ANCOVA finding cannot rule out the possibility that FSIQ contributed to group differences in emotion recognition performance, because the homogeneity of regression assumption was violated (i.e., FSIQ was correlated with performance in the control group only). However, comparisons of IQ-matched groups also confirmed lower accuracy in AgCC group than control group.

**Figure 3:**
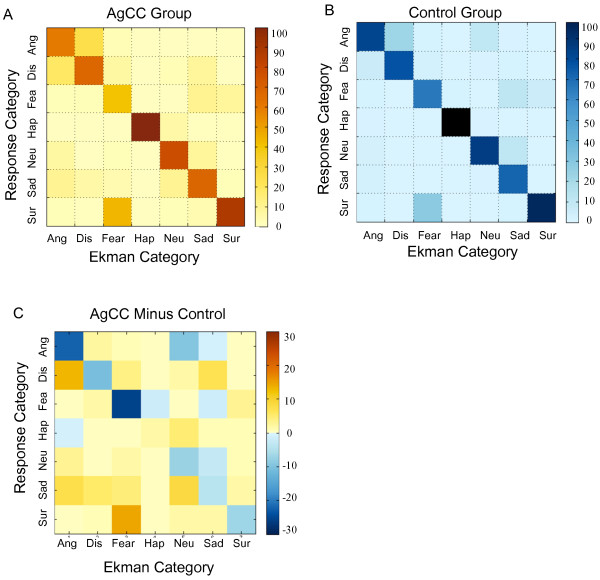
**Average rating matrices by group for emotion recognition tasks.** AgCC **(A)** and control group **(B)** rating matrices. Ekman stimulus classification is on the *x*-axis, and the response category is on the *y*-axis. Each cell represents the relative number of times (*percent*) that a specific response was given for a specific Ekman stimulus category. Concordant ratings fall along the diagonal. **(C)** Difference between the matrices in **(A)** and **(B)** (AgCC group minus control group). *Ang* angry, *Dis* disgust, *Fea* fear, *Hap* happy, *Neu* neutral, *Sur* surprise.

Relative frequency of specific responses to each Ekman category are displayed in Figure 3. Accurate responses fall on the diagonal (top left to bottom right). Both groups tended to mislabel fear as surprise, but did not make the reciprocal error (labeling surprise as fear). Both groups also tended to mislabel disgust as anger, but the reciprocal error (labeling anger as disgust) was only evident in the AgCC group. As indicated by darker blue squares in Figure 3c, the AgCC group was more likely than the control group to mislabel anger as disgust and fear as surprise, while the control group was more likely to accurately identify these emotions (anger, *t*(16) = 2.58, *p* = 0.01, *d* = 1.29, 95% CI 0.07 to infinity (Inf); fear, *t*(16) = 2.62, *p* = 0.009, *d* = 1.31, 95% CI 0.09 to Inf).

**Figure 4:**
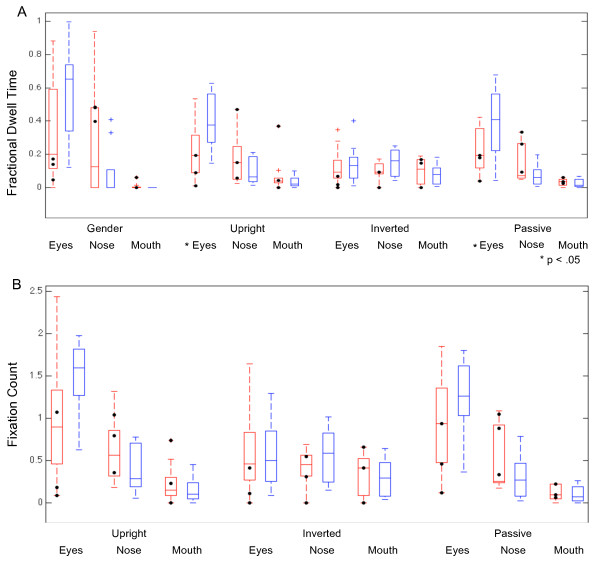
**Eye-tracking results by group for each task and each region of interest.** Fractional dwell time **(A)** by group and region of interest for gender identification, emotion recognition with upright faces, emotion recognition with inverted faces, and passive viewing of faces. Average number of fixations per trial **(B)** by group and region of interest for emotion recognition with upright faces, emotion recognition with inverted faces, and passive viewing of faces. AgCC group in *red* and control group in *blue. Black circles* mark the eye-tracking results of individuals with AgCC who met the current criteria for autism spectrum.

When recognizing emotions in upright faces, the AgCC group exhibited smaller fractional dwell times than the control group in the eye regions (Figure 4a, Table 2), resulting in an interaction of group-by-ROI (*F*(2,48) = 4.95 *p* = 0.011, *η*^2^_*p*_ = 0.17). Post hoc *t* tests confirmed the AgCC group had lower fractional dwell time to eyes *t*(16) = 2.18, *p* = 0.022, *d* = 1.09, 95% CI = 0.036 to Inf, with somewhat larger fractional dwell times to nose (*t*(16) = -1.32, *p* = 0.10, *d* = 0.66, 95% CI = -Inf to 0.024) and mouth (*t*(16) = -1.039, *p* = 0.16, *d* = 0.52, 95% CI –Inf to 0.028). The number of fixations per ROI was generally consistent with findings for fractional dwell time: the AgCC group had fewer fixations than the control group in eye regions and more fixations in mouth and nose regions (Figure 4b).

**Table 2:** Repeated measures ANOVAs for eye-tracking

	**ANOVA**	**ANCOVA**	**FSIQ-matched**
	**( *df *) *F*: *p*, *η***^**2**^_***p***_	**( *df *) *F*: *p*, *η***^**2**^_***p***_	**( *df *) *F*: *p*, *η***^**2**^_***p***_
Gender naming fractional dwell time			
Group	(1,48) 0.06: 0.80, 0.0013	(1,15) 0.008: 0.93, 0.001	(1,36) < 0.001: 0.98, < 0.001
ROI	(2,48) 17.49: < 0.001, 0.42	(1.17,17.6) 0.75: 0.42, 0.048	(2,36) 12.79: < 0.001, 0.42
Interaction	(2,48) 3.97: 0.025, 0.14	(1.17,17.6) 0.60: 0.48, 0.038	(2,36) 0.68: 0.51, 0.036
Upright emotion identification fractional dwell time			
Group	(1,48) 0.34: 0.56, 0.0071	(1,15) 0.317: 0.58, 0.021	(1,36) 0.017: 0.90, < 0.001
ROI	(2,48) 19.13: < 0.001, 0.44	(1.5,21.99) 0.61: 0.50, 0.04	(2,36) 17.28: < 0.001, 0.49
Interaction	(2,48) 4.95: 0.011, 0.17	(1.5,21.99) 1.33: 0.28, 0.08	(2,36) 1.67: 0.20, 0.085
Upright emotion identification fixation count			
Group	(1,48) 0.23: 0.65, 0.005	(1,15) 0.67: 0.43, 0.042	(1,36) 0.16: 0.69, 0.0044
ROI	(2,48) 26.94: < 0.001, 0.53	(1.3,19.6) 0.004: 0.98, 0.0	(2,36) 21.71: < 0.001, 0.55
Interaction	(2,48) 2.98: 0.06, 0.11	(1.3,19.6) 0.97: 0.36, 0.06	(2,36) 0.90: 0.42, 0.048
Inverted emotion identification fractional dwell time			
Group	(1,48) 0.59: 0.44, 0.012	(1,15) 0.033: 0.86, 0.002	(1,36) 0.0017: 0.97, 0.012
ROI	(2,48) 1.38: 0.26, 05	(2,30) 0.18: 0.84, 0.012	(2,36) 0.74: 0.49, 0.039
Interaction	(2,48) 0.53: 0.59, 0.022	(2,30) 0.11: 0.90, 0.007	(2,36) 1.00: 0.38, 0.053
Inverted emotion identification fixation count			
Group	(1,48) 0.08 0.77, 0.002	(1,15) 0.079: 0.78, 0.005	(1,36) 0.01: 0.92, 0.000
ROI	(2,48) 3.22: 0.049, 0.12	(1.5,22.15) 0.098:0.85, 0.006	(2,36) 2.50: 0.096, 0.12
Interaction	(2,48) 0.26: 0.77, 0.01	(1.5,22.15) 0.26: 0.70, 0.02	(2,36) 1.13: 0.33, 0.059
Passive viewing fractional dwell time			
Group	(1,48) 1.07: 0.31, 0.022	(1,15) 0.007: 0.94, 0.000	(1,36) 0.27: 0.61, 0.007
ROI	(2,48) 28.16: < 0.001, 0.54	(2,30) 2.52: 0.098, 0.144	(2,36) 17.32: *p* < 0.001, 0.49
Interaction	(2,48) 5.11: 0.01, 0.18	(2,30) 1.65: 0.21, 0.099	(2,36) 1.44: 0.25, 0.074
Passive viewing fixation count			
Group	(1,48) 0.23: 0.64, 0.005	(1,15) 0.74: 0.40, 0.047	(1,36) 0.098: 0.76, 0.003
ROI	(2,48) = 36.83: < 0.01, 0.61	(1.08,16.1) 0.55: 0.48, 0.04	(2,36) 25.86: < 0.001, 0.59
Interaction	(2,48) 2.71: 0.077, 0.10	(1.08,16.1) 1.01: 0.34, 0.06	(2,36) 0.57: 0.57, 0.031

*ROI* region of interest (eyes, nose, and mouth).

FSIQ was not correlated with fractional dwell time or fixation count in any ROI for either group (Additional file 2: Table S2). However, for both measures, the interaction effect for group-by-ROI was decreased by covarying FSIQ and in the ANOVA with IQ-matched groups (Table 2).

### Emotion recognition: inverted faces

In general, the AgCC group was not markedly worse than the control group at identifying emotions in inverted faces (AgCC = 60.09% ± 14.03, control = 64.95% ± 5.57; Table 1, Figure 2b). However, the group difference varied by emotion, with the AgCC group less accurate than the control group in recognizing anger and fear, and somewhat better at disgust, sadness, and surprise. This resulted in an interaction of group-by-emotion in the ANOVA with 6 emotions, as well as in the ANOVA that included neutral faces (overall accuracy: AgCC = 61.74% ± 30.31, control = 67.66% ± 25.45). One-tailed post hoc *t* tests confirmed that the AgCC group was less accurate than the control group in identifying fear (*t*(16) = 3.22, *p* = 0.003, *d* = 1.61, 95% CI = 0.17 to Inf). There were also less reliable but still notable group differences with anger (*t*(16) = 1.7, *p* = 0.054, *d* = 0.85, 95% CI = -0.004 to Inf) and surprise (*t*(16) = -1.48, *p* = 0.08, *d* = 0.74, 95% CI = -Inf to 0.027).

FSIQ was not correlated with emotion recognition from inverted faces for either group (Additional file 1: Table S1), and covarying FSIQ caused a slight increase in the effect size of the group-by-emotion interaction (both with six and seven emotions, Table 1). Post hoc univariate ANCOVAs confirmed that the AgCC group was less accurate than the control group in identifying inverted fearful faces (*F*(1,15) = 9.60, *p* = 0.007, *η*^2^_*p*_ = 0.39), but covarying FSIQ reduced the effect sizes between groups on other emotions. Effect size for the interaction of group-by-emotion was also slightly larger in comparison of IQ-matched groups than in the entire group (Table 1) and post hoc *t* tests also found the largest group differences for identification of fear (*t*(12) = 3.47, *p* = 0.0023, *d* = 2.00, 95% CI = 0.23 to Inf) and surprise (*t*(12) = -0.249, *p* = 0.014, *d* = 0.44, 95% CI –Inf to -0.068.

When recognizing emotions in inverted faces, the AgCC and control groups exhibited very similar fractional dwell times and fixation counts in each ROI (Table 2; Figure 4). FSIQ was not correlated with fractional dwell time or fixation count in any ROI for either group (Additional file 2: Table S2). No group differences or interactions were found for eye-tracking results when FSIQ was covaried nor in when comparing IQ-matched groups (Table 2).To better understand the effect of face inversion, we examined the difference in accuracy on upright vs. inverted faces at the individual level. For each participant, the percent of correct recognitions for inverted faces was subtracted from those for upright faces, yielding a difference score (see Figure 2c). Facial inversion generally had a negative effect on accuracy for emotion recognition, such that both groups were less accurate for inverted faces compared to upright faces overall (with larger inversion effects on angry, disgusted, sad, and surprised faces). Although the AgCC group was generally less impacted by inversion than the control group on angry, disgusted, fearful, sad, and surprised faces, groups did not differ overall and there was not a group-by-emotion interaction for either the 6-emotion or 7-emotion repeated-measures ANOVA.

However, an interaction of group-by-emotion emerged when FSIQ was covaried and in the ANOVA with IQ-matched groups (Table 1). In post hoc univariate ANCOVAs with each emotion, the AgCC group exhibited a smaller inversion effect than the control group only on surprised faces (*F*(1,15) = 10.98, *p* = 0.005 *η*^2^_*p*_ = 0.423). Similarly, the IQ-matched AgCC group had a smaller inversion effect than control group for surprise (*t*(12) = 3.27, *p* = 0.003 *d* = 1.89, 95% CI 0.15 to Inf).

### Gender recognition: upright faces

The AgCC and control groups did not differ in accuracy of gender recognition (AgCC 97.15% ± 3.84; control 98.41% ± 2.11; *d* = 0.43). Likewise, AgCC and control groups did not differ in gender recognition using univariate ANCOVA nor in the *t* test with IQ-matched groups (*d* = 0.88).

During gender recognition, the AgCC group had lower fractional dwell time in the eye region and higher fractional dwell time in the nose than the control group (Figure 4), resulting in an interaction of group-by-ROI (Table 2; *F*(2,48) = 3.97, *p* = 0.025, *η*^2^_*p*_ = 0.14) and overall effect of ROI. One-tailed post hoc *t* tests were consistent with the overall pattern (eyes *t*(16) = 1.70, *p* = 0.054, 95% confidence interval (CI) = -0.0064 to Inf; nose *t*(16) = -1.54, *p* = 0.072, 95%CI = -Inf to 0.025).

FSIQ was not correlated with fractional dwell time in any ROI for either group (Additional file 2: Table S2), but the group-by-ROI interaction effect was reduced by covarying FSIQ (*F*(1.17,17.6) = 0.60, *p* = 0.48, *η*^2^_*p*_ 0.038) and by using FSIQ-matched groups (*F*(2,36) = 0.68, *p* = 0.51, *η*^2^_*p*_ = 0.036).

### Passive viewing task

On the passive viewing task, the AgCC group had smaller fractional dwell times than the control group in the eye regions and greater fractional dwell times in the nose and mouth regions (Table 2, Figure 4a), resulting in an interaction of group-by-ROI (*F*(2,48) = 5.11, *p* = 0.01, *η*^2^_*p*_ = 0.18). One-tailed post hoc *t* tests confirmed the AgCC group had smaller fractional dwell times in the eye region (*t*(16) = 2.05, *p* = 0.029, *d* = 1.02, 95% CI = 0.025 to Inf) and relatively larger dwell times in the nose (*t*(16) = -1.56, *p* = 0.07, *d* = 0.78, 95% CI = Inf to 0.008), with minimal difference between groups in the mouth region (*t*(16) = -0.42, *p* = 0.34, *d* = 0.21, 95% CI = Inf to 0.015). The pattern of fixations was consistent with fractional dwell time results (Table 2, Figure 4b), with the AgCC group making fewer fixations per trial in the eye regions (*t*(16) = 1.45, *p* = 0.08, *d* = 0.72, 95% CI = -0.07 to Inf) and more fixations in the nose region (*t*(16) = -1.23, *p* = 0.12, *d* = 0.62, 95% CI = -Inf to 0.08), as compared to the control group.

Although FSIQ was not correlated with fractional dwell time or fixation count in any ROI for either group (Additional file 2: Table S2), covarying FSIQ diminished the group-by-ROI interactions for these eye-tracking measures and comparison of IQ-matched groups decreased interaction effects even further (Table 2).

### Impact of autism spectrum diagnosis in AgCC

Consistent with previous studies, approximately 30% of our participants (*n* = 3) with AgCC currently exhibited behavior consistent with a diagnosis of autism spectrum (based on the Autism Diagnostic Observation Schedule and clinical assessment [6]). If autism is an independent, comorbid condition that is not directly related to callosal agenesis, we would expect the subset of individuals with an autism spectrum diagnosis and AgCC to perform most similarly to previous studies of autism. In Figures 2 and 4, scores for these individuals are indicated by black circles. To explore the possibility that individuals with AgCC and autism symptoms were contributing substantially to the findings reported above, we examined the change in effect size when analyses included only the six participants with AgCC who did not meet the autism spectrum criteria (Additional file 3: Tables S3 and Additional file 4: Table S4). Removing participants with AgCC-plus-autism spectrum did not change the pattern of accuracy results for upright or inverted faces. However for upright faces, the group effect of accuracy (*η*^2^_*p*_) was decreased by 0.26 (77%) for 6 emotions and 0.25 (71%) for 6 emotions plus neutral, but effect size did not change notably for gender naming, emotion naming with inverted faces, or the difference in accuracy between upright and inverted (<0.08). Similarly, removing participants with AgCC-plus-autism spectrum did not change the pattern of eye-tracking results for any task, and for all group-related comparisons, the change in effect size was ≤0.1.

### Correlations between eye-tracking and emotion recognition ability

In general, participants with AgCC tended to be less likely than control participants to fixate first within eye region (control mean = 57%; AgCC mean = 31%). We conducted exploratory correlations between accuracy of emotion recognition and two measures of attention to the eyes: latency to first fixation within eye region and fractional dwell time in eye region. In the AgCC group, accuracy was negatively correlated with latency to first fixation within the eye region (*r*_pb_ = -0.07, *t*(824) = -2.02, *p* = 0.02, *d* = -0.14; mean latency on correct responses = 418.24 ms ± 246.44; and incorrect responses = 459.43 ms ± 253.16) and was positively correlated with amount of time looking at the eyes (*r*_pb_ = 0.09, *t*(825) = 2.79, *p* = 0.003, *d* = 0.19). These exploratory analyses suggested that in AgCC, accuracy of emotion recognition may be weakly correlated with visual attention to the eyes, but this is not the case in the control group.

## Discussion

We found that adults with AgCC exhibit impairments in recognizing facial emotions and, furthermore, that these impairments were directly associated with diminished gaze to the eye region in faces. A similar pattern of impairments was seen in the AgCC group when asked to recognize emotions from inverted faces, suggesting the possibility that the deficit may arise at the conceptual or semantic level, rather than be attributable to specific aspects of structural face processing (which should differ for upright vs. inverted faces). Finally, the impairment appeared to be representative of AgCC in general and was not attributable specifically to that subset of subjects who also met the criteria for autism.

### Comparison with other clinical populations

Impaired recognition that is disproportionate for negatively valenced emotions has been reported in a number of clinical samples, including autism [12,40-43] and schizophrenia [55]. As seen in autism and schizophrenia, we found individuals with AgCC had the greatest difficulty in recognizing fearful expressions. Individuals with schizophrenia also struggled with faces displaying disgust [55], but this was not seen in AgCC. Similarly, studies of neurological populations with focal brain lesions have generally pointed to the most severe impairments for fear and variable impairments that are disproportionate for negative emotions [56]. Autism reportedly involves difficulty recognizing both fear and anger [12], a pattern also found in AgCC even after removing individuals with AgCC who also exhibit behavior consistent with autism spectrum. However, the types of errors in AgCC differed from those previously reported in autism [12]. For example, a prior study reported individuals with autism were most likely to mislabel anger as fear (60% of errors) [12], but we found individuals with AgCC most often mislabeled anger as disgust (49% of errors) or sadness (26% of errors). Additionally, they found that individuals with autism mislabeled fear as anger (29%), surprise (42%), and disgust (29%) [12], but in our study, individuals with AgCC primarily mislabeled fear as surprise (73% of errors). The variable impairments in these populations across negatively valenced emotions can be attributed in part to the fact that most tasks yield ceiling levels of accuracy on happiness (cf. Figure 2). It will be important in future studies to attempt to design tasks that equate for the difficulty with which different emotions can be recognized in healthy participants, to then obtain a more unbiased description of differential patterns of impairment across the different emotions.

### Level of processing that might explain the impairment

Several potential explanations may account for impaired emotion recognition in AgCC. Although these explanations are not mutually exclusive, we address each in turn. First, one might hypothesize that this impairment may be due to a general cognitive deficit or a specific deficit in processing facial information. However, in our adults with AgCC, FSIQ was not correlated with emotion recognition or eye-tracking results (Additional file 1: Tables S1 and Additional file 2: Table S2). Although FSIQ in the HC group was positively correlated with emotion recognition, FSIQ did not account for the group difference in recognizing emotions, suggesting that this deficit is not a consequence of general cognitive difficulties. Likewise, intact identification from faces [6] and intact performance on gender identification from faces indicate emotional recognition impairment in AgCC is not due to an impairment in general face-processing ability: they are able to encode, interpret, and verbally label general facial information accurately. Decoupling of face-processing and facial emotion recognition has also been demonstrated in schizophrenia [55] and in autism [57], and is supported by classic double-dissociations in neurological patients with focal brain lesions, indicating that these two skills draw on partly segregated neural substrates.

The second consideration speaks to the level of face-processing strategy that might be compromised in AgCC. The presence of an inversion effect (lower accuracy in recognizing inverted than upright faces) is purported to indicate the use of a normal configural strategy for face processing, whereby the configuration among facial features drives recognition accuracy, rather than identification from any individual facial features (e.g., eyes) in isolation [58-60]. In both the control and AgCC groups, facial inversion generally reduced accuracy of emotion recognition, as expected for configural processing. This prompts the hypothesis that people with AgCC may have impaired facial emotion recognition not so much because they cannot recognize the emotion in the face, but because they cannot translate what they perceive effectively into the verbal labels required in our task. This is consistent with a previously reported dissociation of emotional responsiveness from categorical verbal ratings of emotional scenes [61]. Although skin conductance responses of adults with AgCC discriminated between pleasant, aversive, and neutral images and correlated with arousal ratings (indicating adequate limbic system and right-hemisphere functioning for physiological emotional responses), skin conductance did not correlate with valence ratings. Thus, the impaired emotion recognition in AgCC that we found might be attributable not to impaired structural face processing as such (which may rely largely on right-hemisphere processes), but rather to an effective link between perceptual face representations in the right hemisphere and semantic and conceptual representations that depend on language, in the left hemisphere.

This last consideration implies impaired emotion recognition in AgCC may be a consequence of diminished interhemispheric communication. Social cognition draws substantially on the integration of emotional and nonemotional processing [33,62], modes that are likely to draw differentially upon the right and left hemispheres. Although individuals with AgCC do not exhibit the disconnection syndrome seen in split-brain studies [63-65], effectiveness of interhemispheric communication in AgCC is limited by the complexity of information to be passed between hemispheres. For example, interhemispheric transfer of letters appears to be intact but transfer of information about spatial figures is compromised [66]. However, nothing is known about efficiency with which emotions and emotion labels can be transferred between hemispheres in AgCC. This leaves open the possibility that emotional recognition accuracy is compromised, at least in part, by deficient interhemispheric transfer of information processed locally in the hemispheres. It would be important to test this possibility further in future studies that do not depend on language, for instance face sorting or matching tasks that are entirely visual in nature.

However, we think it likely that the emotion recognition impairments in AgCC also arose, at least to some extent, from abnormal perceptual processing of the faces. The clearest piece of evidence for this is the abnormal eye-tracking data, which features a reduction in fixations and dwell time on the eye region of the face. It is also informative to compare the face inversion effects we found in our AgCC group with face inversion effects reported for other clinical groups. Inversion effects have been found in autism (with a facial identification task, not facial emotion recognition [60]), and in schizophrenia [58,59]. Although face inversion effects in schizophrenia were limited by poor use of configural information in the upright condition, inversion had a relatively greater impact on accuracy for sad and angry faces [58], which were also the most strongly effected in AgCC (albeit less strongly impacted than the control group). This suggests that individuals with AgCC may employ a normal configural strategy for identifying anger and sadness, at least to similar degree as in schizophrenia, but that they do not rely on configural processing as heavily or use it as effectively as controls.

The possibly abnormal configural strategies for facial processing, as well as the abnormal semantic links that we suggested above, may in turn elicit alternate strategies for trying to process the faces—the ones that depend more heavily upon information gathered from specific features. Our eye-tracking abnormalities would broadly support this idea, and importantly, we found a correlation between eye fixations and emotion recognition accuracy. However, it is not possible to determine the causal direction that is driving this correlation: perhaps, people with AgCC have abnormal fixations onto faces because they have difficulty recognizing the emotion; or perhaps, there is a primary attentional deficit revealed by the abnormal fixations that in turn causes the impaired emotion recognition. In schizophrenia, there is a global reduction of face gaze [67], while in autism [12,46] and AgCC, visual attention is allocated in an unusual ratio compared to healthy controls. Relative to controls, both groups spend less time looking at the eyes, while individuals with autism spend more time to looking at the mouths and individuals with AgCC tend to spend slightly more time looking at the nose.

While the utility of atypical facial scanning patterns in autism and AgCC remains uncertain, the similarity of the pattern suggests that interhemispheric integration may play a role in its development and/or maintenance. In AgCC, atypical attention to facial features was evident for all tasks with upright faces (gender identification, emotion recognition, and passive viewing). Thus, reduced attention to the eyes is applied in a task-specific manner, but the negative impact of that strategy is uniquely evident in emotion recognition. Of these tasks, emotion recognition is the most cognitively challenging, and as perceptual and processing load increase, performance is more vulnerable to the processing speed limitations evident in AgCC. Future tasks in which gaze-contingent stimuli are used to control how facial features are fixated experimentally could help to reveal the cause and consequence in the relationship between fixations and emotion recognition.

## Conclusions

In AgCC, impaired emotion recognition is related to a pattern of reduced attention to the eye region. Although impaired emotion recognition may be more notable among individuals with AgCC who also exhibit autism spectrum symptoms, it is also evident in those who do not have such a behavioral diagnosis. Moreover, the presence of atypical facial processing in AgCC does not depend upon the task, the overall intelligence, or the presence of autism spectrum behaviors. We suggest that reduced interhemispheric transfer may be a significant factor in the development and maintenance of impaired facial emotion recognition, perhaps in part through a failure to link perceptual processing of the face to the semantics of the emotion shown. While it remains unclear whether the abnormal eye fixations we also observed are cause or consequence of impaired emotion recognition, they constitute an important biological marker that could also be explored in other clinical populations with disordered emotion recognition.

## Abbreviations

*AgCC*: agenesis of the corpus callosum; *ASD*: autism spectrum disorder; *CI*: confidence interval; *FSIQ*: full-scale intelligence quotient; *Inf*: infinity; *ROI*: region of interest.

## Competing interests

The authors declare that they have no competing interest.

## Authors’ contributions

LP, MS, and RA designed the study. MS, MB, ML, and LP acquired the data. All authors contributed to the data analysis and in writing the paper. Portions of this paper served as the doctoral dissertation of MB, at the Fuller Graduate School of Psychology. All authors read and approved the final manuscript.

## Supplementary Material

Additional file 1: Table S1 Full-scale intelligence quotient and emotion identification. Correlation of full-scale intelligence quotient and accuracy of emotion identification by group. Click here for file

Additional file 2: Table S2 Full-scale intelligence quotient and eye-tracking results. Correlation of full-scale intelligence quotient and eye-tracking results by group. Click here for file

Additional file 3: Table S3 Partial eta squared (accuracy ANOVA). Partial eta squared for accuracy ANOVA: all AgCC vs. controls and AgCC only vs. controls. Click here for file

Additional file 4: Table S4 Effect sizes (eye-tracking ANOVA). Effect sizes (partial eta squared) for eye-tracking ANOVA: all AgCC vs. healthy controls and AgCC only vs. healthy controls. Click here for file
