# Supplementary material for: Facial emotion recognition in agenesis of the corpus callosum
Source: J Neurodev Disord. 2014 Aug 14;6(1):32. doi: 10.1186/1866-1955-6-32 (PMC4335392; doi:10.1186/1866-1955-6-32)
Supplement: Additional file 1: Table S1 — Full-scale intelligence quotient and emotion identification. Correlation of full-scale intelligence quotient and accuracy of emotion identification by group. [file 1866-1955-6-32-S1.doc]

**Additional file 1: Table S1**

Correlation of Full Scale Intelligence Quotient and Accuracy of Emotion Identification by Group

| Upright Emotion Identification | | | | | | |
| --- | --- | --- | --- | --- | --- | --- |
| Group | ROI | r | p-value | 95% confidence interval | | |
| Control | 7 Emotions | 0.76 | * 0.02 | 0.20 | to | 0.95 |
| AgCC |  | -0.10 | 0.79 | -0.72 | to | 0.60 |
| Control | 6 Emotions | 0.71 | * 0.03 | 0.08 | to | 0.93 |
| AgCC |  | -0.30 | 0.43 | -0.80 | to | 0.45 |
| Control | Anger | 0.17 | 0.65 | -0.55 | to | 0.75 |
|  | Disgust | -0.16 | 0.68 | -0.74 | to | 0.56 |
|  | Fear | 0.37 | 0.32 | -0.39 | to | 0.83 |
|  | Happy | 0.28 | 0.46 | -0.47 | to | 0.80 |
|  | Neutral | 0.73 | * 0.02 | 0.14 | to | 0.94 |
|  | Sad | 0.15 | 0.71 | -0.57 | to | 0.74 |
|  | Surprise | 0.18 | 0.64 | -0.55 | to | 0.75 |
| AgCC | Anger | -0.37 | 0.33 | -0.83 | to | 0.39 |
|  | Disgust | 0.49 | 0.18 | -0.26 | to | 0.87 |
|  | Fear | 0.32 | 0.40 | -0.44 | to | 0.81 |
|  | Happy | NaN | NaN | NaN | to | NaN |
|  | Neutral | 0.44 | 0.23 | -0.31 | to | 0.86 |
|  | Sad | -0.28 | 0.47 | -0.80 | to | 0.47 |
|  | Surprise | -0.07 | 0.86 | -0.70 | to | 0.62 |
| Inverted Emotion Identification | | | | | | |
| Group | ROI | r | p-value | 95% confidence interval | | |
| Control | 7 Emotions | 0.14 | 0.72 | -0.58 | to | 0.74 |
| AgCC |  | 0.40 | 0.29 | -0.36 | to | 0.84 |
| Control | 6 Emotions | -0.41 | 0.27 | -0.84 | to | 0.35 |
| AgCC |  | 0.44 | 0.23 | -0.31 | to | 0.86 |
| Control | Anger | -0.45 | 0.23 | -0.86 | to | 0.31 |
|  | Disgust | -0.06 | 0.88 | -0.70 | to | 0.63 |
|  | Fear | 0.35 | 0.36 | -0.41 | to | 0.82 |
|  | Happy | 0.26 | 0.49 | -0.49 | to | 0.79 |
|  | Neutral | 0.29 | 0.46 | -0.47 | to | 0.80 |
|  | Sad | -0.39 | 0.30 | -0.84 | to | 0.37 |
|  | Surprise | 0.02 | 0.96 | -0.65 | to | 0.68 |
| AgCC | Anger | 0.12 | 0.76 | -0.59 | to | 0.73 |
|  | Disgust | 0.39 | 0.30 | -0.37 | to | 0.84 |
|  | Fear | -0.57 | 0.11 | -0.89 | to | 0.16 |
|  | Happy | 0.56 | 0.12 | -0.17 | to | 0.89 |
|  | Neutral | 0.00 | 1.00 | -0.66 | to | 0.66 |
|  | Sad | -0.17 | 0.67 | -0.75 | to | 0.56 |
|  | Surprise | 0.63 | 0.07 | -0.06 | to | 0.91 |

*Note:* p < .05; AgCC = Agenesis of the corpus callosum group.
