# Supplementary material for: Facial emotion recognition in agenesis of the corpus callosum
Source: J Neurodev Disord. 2014 Aug 14;6(1):32. doi: 10.1186/1866-1955-6-32 (PMC4335392; doi:10.1186/1866-1955-6-32)
Supplement: Additional file 2: Table S2 — Full-scale intelligence quotient and eye-tracking results. Correlation of full-scale intelligence quotient and eye-tracking results by group. [file 1866-1955-6-32-S2.doc]

**Additional file 2: Table S2**

Correlation of Full Scale Intelligence Quotient and Eye-Tracking Results by Group

| Group | ROI | r | p-value | 95% confidence interval | | |
| --- | --- | --- | --- | --- | --- | --- |
| Gender Naming Fractional Dwell Time | | | | | | |
| Control | Eyes | 0.31 | 0.41 | -0.44 | to | 0.81 |
|  | Nose | 0.30 | 0.44 | -0.46 | to | 0.80 |
|  | Mouth | NaN | NaN | NaN | to | NaN |
| AgCC | Eyes | 0.31 | 0.41 | -0.44 | to | 0.81 |
|  | Nose | -0.34 | 0.37 | -0.82 | to | 0.42 |
|  | Mouth | -0.52 | 0.15 | -0.88 | to | 0.22 |
| Upright Emotion Identification Fractional Dwell Time | | | | | | |
| Control | Eyes | 0.19 | 0.62 | -0.54 | to | 0.76 |
|  | Nose | -0.18 | 0.65 | -0.75 | to | 0.55 |
|  | Mouth | -0.27 | 0.49 | -0.79 | to | 0.48 |
| AgCC | Eyes | 0.32 | 0.41 | -0.44 | to | 0.81 |
|  | Nose | 0.23 | 0.56 | -0.52 | to | 0.77 |
|  | Mouth | -0.52 | 0.15 | -0.88 | to | 0.22 |
| Upright Emotion Identification Fixation Count | | | | | | |
| Control | Eyes | -0.27 | 0.49 | -0.79 | to | 0.48 |
|  | Nose | -0.25 | 0.51 | -0.78 | to | 0.50 |
|  | Mouth | -0.27 | 0.48 | -0.79 | to | 0.48 |
| AgCC | Eyes | 0.20 | 0.61 | -0.54 | to | 0.76 |
|  | Nose | -0.02 | 0.95 | -0.68 | to | 0.65 |
|  | Mouth | -0.43 | 0.25 | -0.85 | to | 0.33 |
| Inverted Emotion Identification Fractional Dwell Time | | | | | | |
| Control | Eyes | 0.09 | 0.81 | -0.61 | to | 0.71 |
|  | Nose | 0.03 | 0.94 | -0.65 | to | 0.68 |
|  | Mouth | -0.21 | 0.59 | -0.77 | to | 0.53 |
| AgCC | Eyes | 0.24 | 0.54 | -0.51 | to | 0.78 |
|  | Nose | 0.50 | 0.17 | -0.24 | to | 0.88 |
|  | Mouth | 0.03 | 0.94 | -0.65 | to | 0.68 |
| Inverted Emotion Identification Fixation Count | | | | | | |
| Control | Eyes | -0.11 | 0.78 | -0.72 | to | 0.60 |
|  | Nose | -0.29 | 0.46 | -0.80 | to | 0.47 |
|  | Mouth | -0.40 | 0.29 | -0.84 | to | 0.36 |
| AgCC | Eyes | 0.17 | 0.66 | -0.56 | to | 0.75 |
|  | Nose | 0.24 | 0.54 | -0.51 | to | 0.78 |
|  | Mouth | -0.06 | 0.88 | -0.69 | to | 0.63 |
| Passive Viewing Fractional Dwell Time | | | | | | |
| Control | Eyes | 0.41 | 0.27 | -0.35 | to | 0.85 |
|  | Nose | -0.56 | 0.12 | -0.89 | to | 0.17 |
|  | Mouth | -0.57 | 0.11 | -0.90 | to | 0.15 |
| AgCC | Eyes | 0.11 | 0.77 | -0.60 | to | 0.72 |
|  | Nose | -0.11 | 0.78 | -0.72 | to | 0.60 |
|  | Mouth | -0.30 | 0.44 | -0.80 | to | 0.46 |
| Passive Viewing Fixation Count | | | | | | |
| Control | Eyes | 0.26 | 0.50 | -0.49 | to | 0.79 |
|  | Nose | -0.55 | 0.12 | -0.89 | to | 0.18 |
|  | Mouth | -0.47 | 0.20 | -0.87 | to | 0.28 |
| AgCC | Eyes | 0.20 | 0.60 | -0.53 | to | 0.76 |
|  | Nose | -0.23 | 0.56 | -0.77 | to | 0.51 |
|  | Mouth | -0.12 | 0.75 | -0.73 | to | 0.59 |

*Note:* AgCC = Agenesis of the corpus callosum group; NaN = Not a number, indicating analysis was not possible.
