# Supplementary material for: Facial emotion recognition in agenesis of the corpus callosum
Source: J Neurodev Disord. 2014 Aug 14;6(1):32. doi: 10.1186/1866-1955-6-32 (PMC4335392; doi:10.1186/1866-1955-6-32)
Supplement: Additional file 3: Table S3 — Partial eta squared (accuracy ANOVA). Partial eta squared for accuracy ANOVA: all AgCC vs. controls and AgCC only vs. controls. [file 1866-1955-6-32-S3.doc]

**Additional file 3: Table S3**

Partial eta squared for Accuracy ANOVA: All AgCC vs. Healthy Controls and AgCC-Only vs. Healthy Controls

|  | All AgCC | AgCC-only | Difference |
| --- | --- | --- | --- |
| Upright Faces: Accuracy for 6 Emotions | | | |
| Group | ** .39 | ** .13 | - .26 |
| Emotion | ** .55 | ** .50 | -.05 |
| Interaction | .11 | .09 | -.02 |
| Upright Faces: Accuracy for 7 Emotions | | | |
| Group | ** .35 | ** .10 | -.25 |
| Emotion | **.51 | ** .48 | -.03 |
| Interaction | .10 | .09 | -.01 |
| Inverted Faces: Accuracy for 6 Emotions | | | |
| Group | .055 | < .001 | - .054 |
| Emotion | ** .53 | ** .48 | -.05 |
| Interaction | ** .20 | ** .23 | .03 |
| Inverted Faces: Accuracy for 7 Emotions | | | |
| Group | .08 | < .001 | - .079 |
| Emotion | **.51 | ** .46 | -.05 |
| Interaction | **.18 | ** .20 | .02 |
| Upright Accuracy Minus Inverted Accuracy for 6 Emotions | | | |
| Group | .12 | ** .11 | -.01 |
| Emotion | ** .26 | ** .21 | -.05 |
| Interaction | .08 | * .13 | .5 |
| Upright Accuracy Minus Inverted Accuracy for 6 Emotions | | | |
| Group | .10 | *.06 | -.04 |
| Emotion | ** .25 | ** .19 | -.06 |
| Interaction | .08 | * .15 | .07 |

*Note:* * p > 05; ** p > .01. AgCC = Agenesis of the corpus callosum.
