# Supplementary material for: Facial emotion recognition in agenesis of the corpus callosum
Source: J Neurodev Disord. 2014 Aug 14;6(1):32. doi: 10.1186/1866-1955-6-32 (PMC4335392; doi:10.1186/1866-1955-6-32)
Supplement: Additional file 4: Table S4 — Effect sizes (eye-tracking ANOVA). Effect sizes (partial eta squared) for eye-tracking ANOVA: all AgCC vs. healthy controls and AgCC only vs. healthy controls. [file 1866-1955-6-32-S4.doc]

**Additional file 4: Table S4**

Effect Sizes (partial eta squared) for Eye-tracking ANOVA: All AgCC vs. Healthy Controls and AgCC-Only vs. Healthy Controls

|  | All AgCC | AgCC only | Difference |
| --- | --- | --- | --- |
| Gender Naming Fractional Dwell Time | | | |
| Group | .001 | < .001 | 0 |
| ROI | ** .42 | ** .48 | .06 |
| Interaction | * .14 | .04 | -.10 |
| Upright Emotion Identification Fractional Dwell Time | | | |
| Group | .007 | .006 | - .001 |
| ROI | ** .44 | ** .59 | .15 |
| Interaction | * .17 | .09 | -.08 |
| Upright Emotion Identification Fixation Count | | | |
| Group | .005 | < .001 | -.004 |
| ROI | ** .53 | ** .63 | .10 |
| Interaction | .11 | .03 | -.08 |
| Inverted Emotion Identification Fractional Dwell Time | | | |
| Group | .012 | < .001 | -.011 |
| ROI | .05 | .12 | .07 |
| Interaction | .02 | .02 | 0 |
| Inverted Emotion Identification Fixation Count | | | |
| Group | .002 | .01 | -.008 |
| ROI | *.12 | ** .22 | .10 |
| Interaction | .01 | .04 | .03 |
| Passive Viewing Fractional Dwell Time | | | |
| Group | .022 | .023 | .001 |
| ROI | ** .54 | ** .59 | .05 |
| Interaction | * .18 | .08 | -.10 |
| Passive Viewing Fixation Count | | | |
| Group | .005 | < .001 | -.004 |
| ROI | * .61 | ** .69 | .08 |
| Interaction | .10 | .02 | -.08 |

*Note:* * p > 05; ** p > .01. AgCC = Agenesis of the corpus callosum.
